# Unique microbial communities persist in individual cystic fibrosis patients throughout a clinical exacerbation

**DOI:** 10.1186/2049-2618-1-27

**Published:** 2013-11-01

**Authors:** Katherine E Price, Thomas H Hampton, Alex H Gifford, Emily L Dolben, Deborah A Hogan, Hilary G Morrison, Mitchell L Sogin, George A O’Toole

**Affiliations:** 1 Department of Microbiology and Immunology, Geisel School of Medicine at Dartmouth, Hanover, NH 03755, USA; 2 Dartmouth-Hitchcock Medical Center, Section of Pulmonary and Critical Care Medicine, Lebanon 03756, New Hampshire, USA; 3 Josephine Bay Paul Center for Comparative Molecular Biology and Evolution, Marine Biological Laboratory, Woods Hole, MA 02543, USA

**Keywords:** Cystic fibrosis, Cystic fibrosis pulmonary exacerbation, Microbiome, Sputum, *Pseudomonas aeruginosa*

## Abstract

**Background:**

Cystic fibrosis (CF) is caused by inherited mutations in the cystic fibrosis transmembrane conductance regulator gene and results in a lung environment that is highly conducive to polymicrobial infection. Over a lifetime, decreasing bacterial diversity and the presence of *Pseudomonas aeruginosa* in the lung are correlated with worsening lung disease. However, to date, no change in community diversity, overall microbial load or individual microbes has been shown to correlate with the onset of an acute exacerbation in CF patients. We followed 17 adult CF patients throughout the course of clinical exacerbation, treatment and recovery, using deep sequencing and quantitative PCR to characterize spontaneously expectorated sputum samples

**Results:**

We identified approximately 170 bacterial genera, 12 of which accounted for over 90% of the total bacterial load across all patient samples. Genera abundant in any single patient sample tended to be detectable in most samples. We found that clinical stages could not be distinguished by absolute *Pseudomonas aeruginosa* load, absolute total bacterial load or the relative abundance of any individual genus detected, or community diversity. Instead, we found that the microbial structure of each patient’s sputum microbiome was distinct and resilient to exacerbation and antibiotic treatment.

**Conclusion:**

Consistent with previously reported sputum microbiome studies we found that total and relative abundance of genera at the population level were remarkably stable for individual patients regardless of clinical status. Patient-by-patient analysis of diversity and relative abundance of each individual genus revealed a complex microbial landscape and highlighted the difficulty of identifying a universal microbial signature of exacerbation. Overall, at the genus level, we find no evidence of a microbial signature of clinical stage.

## Background

Cystic fibrosis (CF) is a human genetic disorder caused by mutations in the cystic fibrosis transmembrane conductance regulator (CFTR) gene. Mutations in the CFTR gene lead to decreases in periciliary fluid layer and increased volume and viscosity of mucus in the lungs, resulting in an environment highly conducive to microbial growth. The lungs of CF patients eventually become permanently colonized and chronically inflamed leading to gradual pulmonary function decline and the bulk of CF-associated mortality [1-5]. This inflammatory process includes acute CF pulmonary exacerbations (CFPEs) characterized by decreased lung function, changes in cough, sputum production, shortness of breath, decreased energy level and appetite, and weight loss [6]. Patients experiencing a CFPE typically require hospitalization and receive intravenous antibiotics chosen based on the antibiotic resistance profiles of *Pseudomonas aeruginosa*.

Conventional culturing techniques using sputum or bronchial alveolar lavage (BAL) samples have identified several key microbes that contribute to CF lung infection and disease progression. These techniques have revealed that CF lung colonization generally begins early in life with *Staphylococcus aureus* and *Haemophilus influenzae*, which are later supplanted by *P. aeruginosa*. This change in microbial colonizers, specifically the appearance of *P. aeruginosa*, is associated with decreasing lung function [7-9]. Advances in culture-independent, deep-sequencing technology have revealed that the landscape of microbes in the CF lung is much richer than previously appreciated. Deep sequencing of the CF-derived sputum and BAL samples has revealed that there are dozens of bacterial genera present, including *Streptococcus* and a variety of anaerobes including *Prevotella, Fusobacterium* and *Veillonella*[10-20]. The relative contribution by these newly identified members of the CF airway microbiome to clinical status and disease progression, however, is still unclear. Total bacterial load (or load of *Pseudomonas* in particular) has been reported to be a poor predictor of exacerbations [21]. Previous reports have suggested a role for bacterial diversity as a determinant of clinical stability over the long term [11,16-20,22]. It is unclear whether increased diversity is directly beneficial to the patient or if increased diversity correlates with stable disease because stable patients experience fewer exacerbations and therefore fewer exposures to antibiotics. Whether the effect is direct or indirect, thus far, bacterial diversity has not been shown to predict the onset of an exacerbation [11,19].

Here, we build on these studies, testing the hypothesis that there is a microbial signature of CF exacerbations. To identify such a microbial signature of CF exacerbations, we followed the short-term microbiome dynamics both at the community population level and organism-by-organism in an independent cohort of adult CF patients. Although we detected over 170 bacterial genera, 12 genera account for approximately 90% of the bacterial diversity across all samples, consistent with our finding that high abundance in a single sample is correlated with prevalence across all samples. Genera that thrive in a single patient tend to be present in most patients. We found that patient microbial communities were highly distinct and observed remarkable stability and resilience throughout the exacerbation cycle. We analyzed short-term microbiome dynamics, organism-by-organism, as a function of transition from one clinical state to the next, and found no evidence of a microbial signature of exacerbation, consistent with several previous studies that measured the population dynamics of the sputum microbiome [11,19,23]. Overall, we found that the microbial communities in the sputum of individual CF patients are both distinct and resilient throughout the stresses of an exacerbation and antibiotic treatment.

## Methods

### Patient cohort, sample collection and genomic DNA preparation

As described by Gifford *et al.*[24], 17 adult CF patients were recruited to the study. The Committee for the Protection of Human Subjects (CPHS) at Dartmouth College approved the sputum collection protocol (CPHS #22506) and all patients provided written informed consent prior to participating in the study. Patient ages, FEV_1_, antibiotics administered at exacerbation and dates of sample collection are listed in Additional file 1: Table S1.

Genomic DNA was isolated from spontaneously expectorated sputum samples produced at four clinically defined stages: baseline, exacerbation, treatment and recovery (BETR). Baseline samples were collected at a routine, quarterly clinic visit. Baseline samples are clinically defined and include those that were collected before an exacerbation occurred and after a full recovery from an exacerbation. Exacerbation samples were obtained less than 24 hours after CFPE determination following admittance to the hospital. Treatment samples were collected less than 24 hours prior to completing hospital stay where patients received intravenous (IV) antibiotics chosen based on their clinical laboratory history of *Pseudomonas* susceptibility and resistance profiles. Recovery samples were taken at the next routine, quarterly visit to the clinic. It should be noted that exacerbation sputum samples include samples that were taken before and after the administration of IV antibiotics, but always within 24 hours of hospital admittance. Of the seventeen patients analyzed in this study, we considered nine of these patient sputum sample sets complete and eight incomplete. Table 1 shows absolute bacterial abundance (copies 16s rRNA/gram sputum). Six patients produced sputum at all four BETR stages, and three patients produced sputum at the baseline, exacerbation and recovery (BER) stages but no sputum at the treatment stage, as their condition improved such that they did not produce sputum at that time point. The remaining eight patients were lost to follow-up and/or have incomplete datasets due to missed visits or late recruitment. For each analysis below, the datasets used are indicated.

**Table 1:** Absolute abundance of total bacterial load in each sample

**Patient ID**	**Baseline**	**Exacerbation**	**Treatment**	**Recovery**
100	4.36 × 10^+9^	3.71 × 10^+10^	2.16 × 10^+9^	2.40 × 10^+10^
101	6.73 × 10^+10^	8.97 × 10^+10^	6.15 × 10^+9^	6.60 × 10^+10^
200	4.02 × 10^+10^	1.45 × 10^+10^	2.45 × 10^+8^	1.07 × 10^+11^
204	5.73 × 10^+10^	5.23 × 10^+9^	1.78 × 10^+10^	8.42 × 10^+6^
207	4.10 × 10^+10^	1.23 × 10^+9^	1.41 × 10^+10^	1.60 × 10^+10^
212	4.02 × 10^+9^	1.99 × 10^+10^	1.61 × 10^+10^	1.30 × 10^+10^
201	1.38 × 10^+10^	2.59 × 10^+10^	n.s.	1.88 × 10^+10^
202	2.39 × 10^+11^	5.65 × 10^+10^	n.s.	1.24 × 10^+10^
205	9.37 × 10^+9^	1.51 × 10^+10^	n.s.	3.19 × 10^+9^
102	5.09 × 10^+10^	n.s.	n.s.	n.s
203	9.98 × 10^+9^	1.29 × 10^+10^	n.s.	n.s.
206	n.s.	n.s.	5.33 × 10^+9^	1.17 × 10^+10^
208	n.s.	1.07 × 10^+10^	n.s.	n.s.
209	1.53 × 10^+10^	n.s.	n.s.	n.s.
210	n.s.	2.79 × 10^+10^	1.68 × 10^+10^	n.s.
211	5.70 × 10^+10^	3.66 × 10^+9^	n.s.	n.s.
213	n.s.	4.32 × 10^+10^	9.30 × 10^+10^	n.s.

n.s, no sample.

### Community analyses

Genomic DNA (gDNA) was isolated from patient sputum samples as previously described [18] for use in 454 pyrosequencing of the V4-V6 regions of the 16S rRNA gene, and quantitative PCR (qPCR) analysis of total bacterial load and relative abundance of *P. aeruginosa*. Briefly, gDNA was isolated with a modified protocol of the Gentra PureGeneYeast/Bact. Kit. Patient sputum samples were weighed and their mass noted before sputum samples were resuspended and diluted twofold to fivefold in Tris-EDTA (TE) + 0.08% dithiothreitol (DTT). Diluted samples were passed repeatedly through syringes with 16, 20 and 23 gauge needles until homogeneous. Homogenates were treated with a final concentration of 3 mg/mL lysozyme for 30 minutes at 37°C. Samples were then incubated in cell lysis buffer (Gentra) for 15 minutes at 80°C. gDNA from these treated samples was isolated following the manufacturer’s protocol. The resulting gDNA was used in deep sequencing and qPCR analysis. gDNA for qPCR controls was prepared using the Gentra PureGene Yeast/Bact. Kit according to the manufacturer’s instructions for Gram-positive or Gram-negative species as appropriate.

Deep sequencing, bioinformatic quality filtering and operational taxonomic unit assignments were performed as previously described [18]. Briefly, a custom bioinformatics pipeline at the Marine Biological Laboratory performed quality filtering to remove low quality reads (average quality scores less than 30) and sequences lacking exact primer matches or containing ambiguous bases (Ns). Chimerical reads were removed using the UChime algorithm, which combines the *de novo* and reference database modes of ChimeraSlayer GOLD. A taxonomy was assigned to each unique read by the GAST algorithm; UCLUST identified the operational taxonomic units with 97% sequencing identity. Individual reads, taxon assignments and descriptions of individual clusters are accessible on the website Visualization and Analysis of Microbial Population Structures [25] and the NCBI website [26], SRA study accession number SRP025173.

The absolute abundance of the total bacterial load per gram of sputum sample was determined by qPCR using methods similar to those previously published [11,21] with universal primers to the 16S rRNA gene originally described in Maeda *et al*., 2003 and evaluated in Horz *et al*., 2005 for broad range amplification of bacterial species (Universal For/Rev 5′-GTGSTGCAYGGYTGTCGTCA-3′/5′-ACGTCRTCCMCACCTTCCTC-3′) [27,28]. The number of 16S molecules in a given 10 ng qPCR reaction was multiplied by the total number of nanograms in the entire sputum gDNA prep and then divided by the sputum mass at the time of collection to give an absolute abundance of 16S molecules/gram sputum.

The relative abundance of *P. aeruginosa* was determined as previously described [18] using primers to the *P. aeruginosa* housekeeping gene *rplU* (*rplU* For 5′-GCAGCACAAAGTCACCGAAGG-3′ and *rplU* Rev 5′-CCGTGGGAAACCACTTCAGC-3′) and universal primers Universal For/Rev.

### Statistical analyses

All statistical analyses were performed on taxa at the level of genus normalized by the percentage within the datasets where the frequency of each taxonomic assignment was reported as a percentage (number of reads assigned to a taxonomy over total number of reads in the dataset). Heat maps were developed using complete hierarchical clustering by Euclidian distance, using the heatmap.2 function in R as implemented in gplots [29]. Principal coordinate analysis was performed using the prcomp routine in pcaComp in R [30]. The Simpson diversity index was calculated at the genus level using the diversity function in the R package vegan [31]. Mixed-effect linear models with clinical stage as a categorical fixed effect and patient as a random effect were generated in R to estimate diversity as a function of clinical status using the lme package in R.

## Results

### Bacterial load in sputum does not correlate with clinical stage

**Figure 1:**
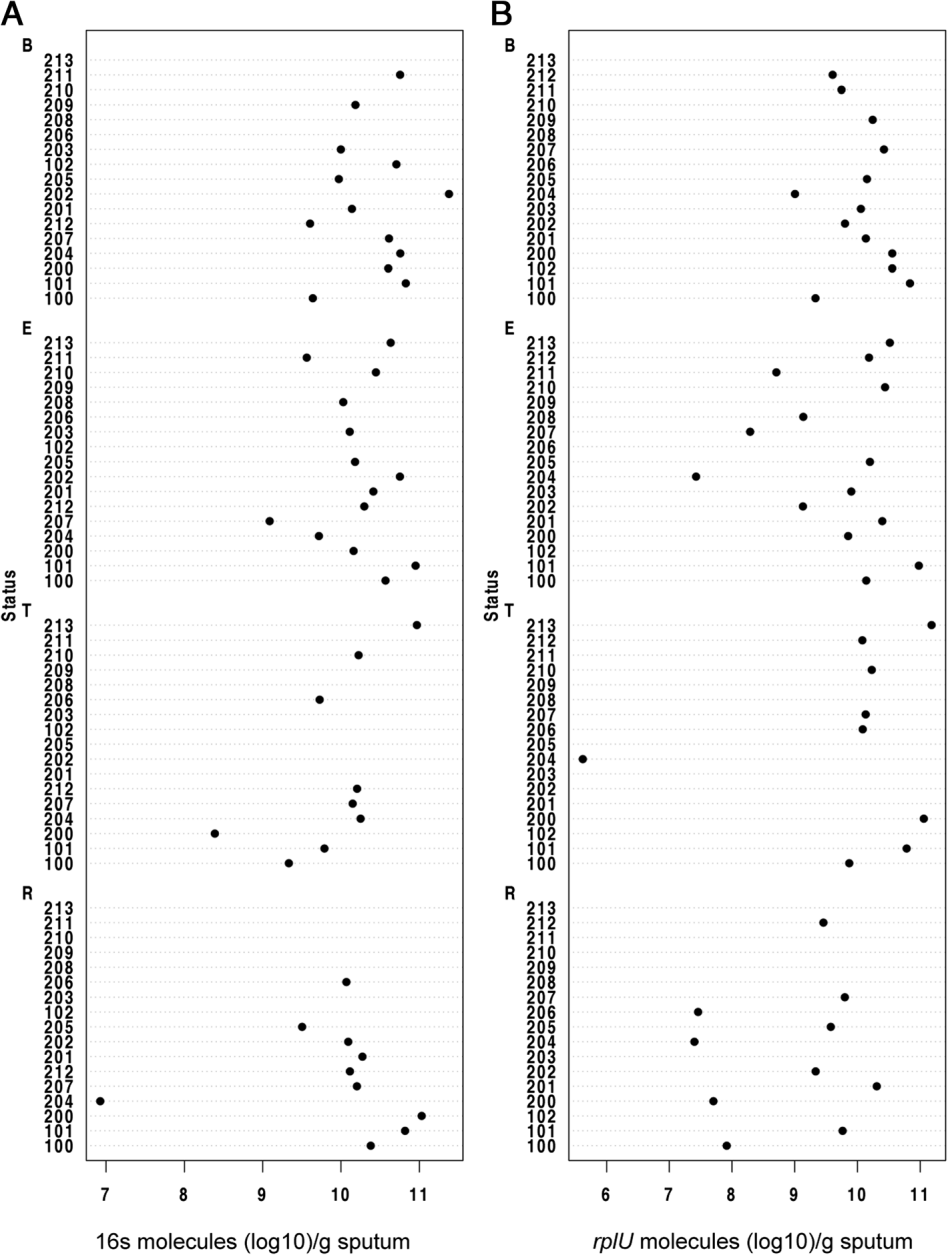
**Neither absolute bacterial load nor absolute abundance of *Pseudomonas *correlates with clinical stage. (A)** Absolute bacterial load in each sputum sample calculated by qPCR with universal primers to 16s rRNA gene normalized to gram of sputum extracted for this analysis (16s molecules (log_10_)/gram sputum). **(B)** Absolute abundance of *P. aeruginosa* in each sputum sample calculated by qPCR with primers to *rplU*, a reference gene validated as specific to *P. aeruginosa*, normalized to gram of sputum extracted for this analysis (*rplU* molecules (log_10_)/gram sputum). B, baseline; E, exacerbation; R, recovery; T, treatment.

Bruce and colleagues reported that the absolute abundance of *Pseudomonas* and other bacteria did not change during the three weeks before an exacerbation occurs [21]. We extend this work using similar methods to include all clinical stages of an exacerbation (baseline, exacerbation, treatment and recovery) in a larger, independent cohort. We measured the total bacterial abundance and the abundance of *Pseudomonas* by qPCR normalized to sputum weight. The total bacterial load per gram of sputum in all samples analyzed ranged from 8.42 × 10^6^ to 2.39 × 10^11^ (Table 1, Figure 1A). The total bacterial load (defined as the number of 16S molecules/gram sputum) as well as the absolute abundance of *Pseudomonas* (defined as the number of *rplU* molecules/gram sputum), remained relatively stable throughout all clinical stages, and ANOVA of the log-transformed data revealed no significant difference among clinical stages for total bacterial load (Figure 1A) or *Pseudomonas* absolute abundance (Figure 1B). For example, as shown in Table 1, six out of eleven patients with available measurements at both time points saw an increased bacterial load as they made the transition from baseline (B) to exacerbation (E), but five out of eleven patients saw a decrease, consistent with what one would expect by chance. Similarly, for the nine patients with measurements during exacerbation (E) that had available measurements at recovery (R), there was no significant difference (*P* = 0.25). In summary, data from spontaneously expectorated sputum support neither the hypothesis that increases in bacterial load precipitate exacerbation nor the hypothesis that recovery is achieved by a reduction in bacterial load.

### Twelve highly prevalent genera account for most of the sputum communities in our cohort

**Figure 2:**
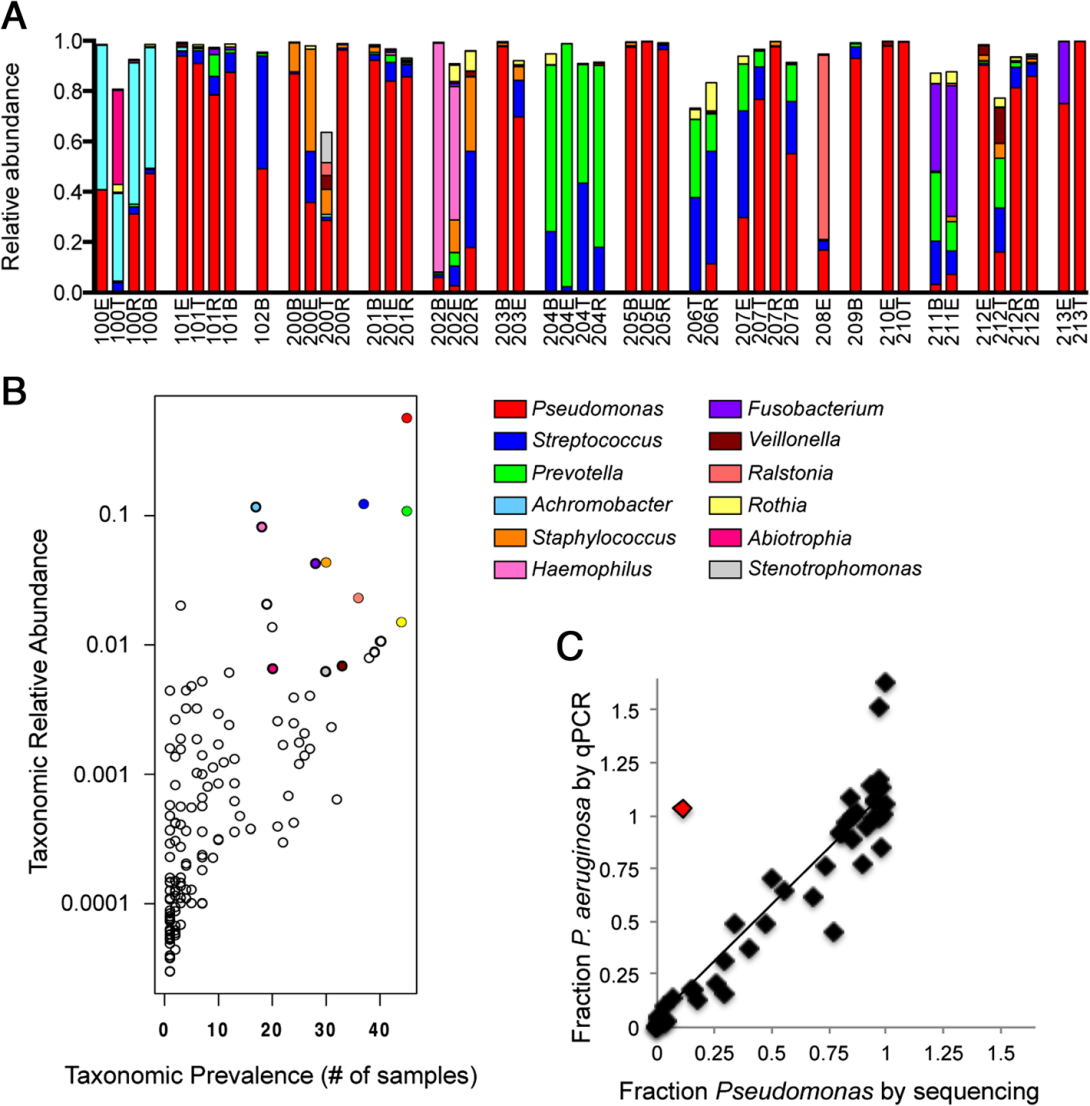
**Relative abundance of top twelve genera. (A)** Stacked bar charts of relative abundance (left *y*-axis) of the top 12 genera for each patient across a clinical exacerbation show that 12 genera explain 90% of the complexity for all patient samples. **(B)** Relative abundance of the top 12 genera are plotted against the prevalence of each genus in the nine complete (BETR, BER) patient samples and show that bacteria that are highly abundant in a single patient are also highly prevalent across patients. The colored dots indicate those genera that are both highly abundant and highly prevalent, and the colors correspond to the legend shown in panel A. Colors in panels A and B correspond to genera as indicated by the legend beneath panel A. **(C)** The fraction of *P. aeruginosa* determined by qPCR (*rplU* detection/16s rRNA detection) correlates to the fraction of deep-sequencing reads assigned to the *Pseudomonas* genus. qPCR samples were analyzed six times and the median fraction values for each sample are shown. There is one outlier in this dataset (sample 206R, shown in red). When this outlier is removed from the analysis, the linear regression slope is 1.1 and *R*^2^ = 0.90. BER, baseline, exacerbation and recovery; BETR, baseline, exacerbation, treatment and recovery; qPCR, quantitative PCR.

We and others have previously reported that the sputum microbiome includes genera other than *Pseudomonas*[11,18-20,23]. To characterize the prevalence and abundance of bacterial genera in spontaneously expectorated sputum, genomic DNA was isolated from all samples and the V6-V4 region of the 16S rRNA gene was sequenced by 454 pyrosequencing, as previously described [18]. The complete sputum communities and the relative abundance for each genus for all patient samples are listed in Additional file 2: Table S2. In all, over 170 bacterial genera were detected in these sputum samples. Despite this large number of organisms, a relatively small collection of genera account for 90% of the reads in all samples. Figure 2A shows the relative abundance of the top 12 genera (*Pseudomonas*, *Streptococcus*, *Prevotella*, *Achromobacter*, *Staphylococcus*, *Haemophilus*, *Fusobacterium*, *Veillonella, Ralstonia*, *Rothia*, *Abiotrophia* and *Stenotrophomonas*) found in our patient cohort.

### Relative bacterial abundance within a patient is correlated with prevalence across all patients

We found a positive correlation between relative abundance and prevalence of bacteria in the CF sputum. That is, a genus that was highly abundant in one sample was also highly prevalent across all samples (Figure 2B). For example, *Pseudomonas* (red dot, upper right) was found in 45 of 46 samples with a mean relative abundance of 0.55 when present. The three genera with the highest relative abundance and prevalence were *Pseudomonas*, *Streptococcus* and *Prevotella*, genera previously reported to be abundant in other patient cohorts [11,18-20,23].

### qPCR analysis validates deep-sequencing measurements of *Pseudomonas*

We sought to confirm the pyrosequencing results by an independent method. Because the presence of *P. aeruginosa* is associated with worsening lung function [7-9] and *Pseudomonas* is found in the majority of our patients, the relative abundance of *P. aeruginosa* was measured by qPCR with primers specific to the *P. aeruginosa* reference gene *rplU*, which were previously validated as specific for *P. aeruginosa*[18]. The relative abundance of *P. aeruginosa* as measured by qPCR (median of six technical replicates) was plotted versus the relative abundance of *Pseudomonas* as determined by deep sequencing (Figure 2C) for all 17 patients. We observed a strong correlation between pyrosequencing and qPCR data with the exception of one outlier (Figure 2C, red diamond). Excluding this outlier from the analysis yields a regression slope of 1.1 with a correlation coefficient of 0.90, indicating that our qPCR findings validate our pyrosequencing results.

### Microbial communities are resilient throughout clinical exacerbations and cluster by patient not by clinical stage

**Figure 3:**
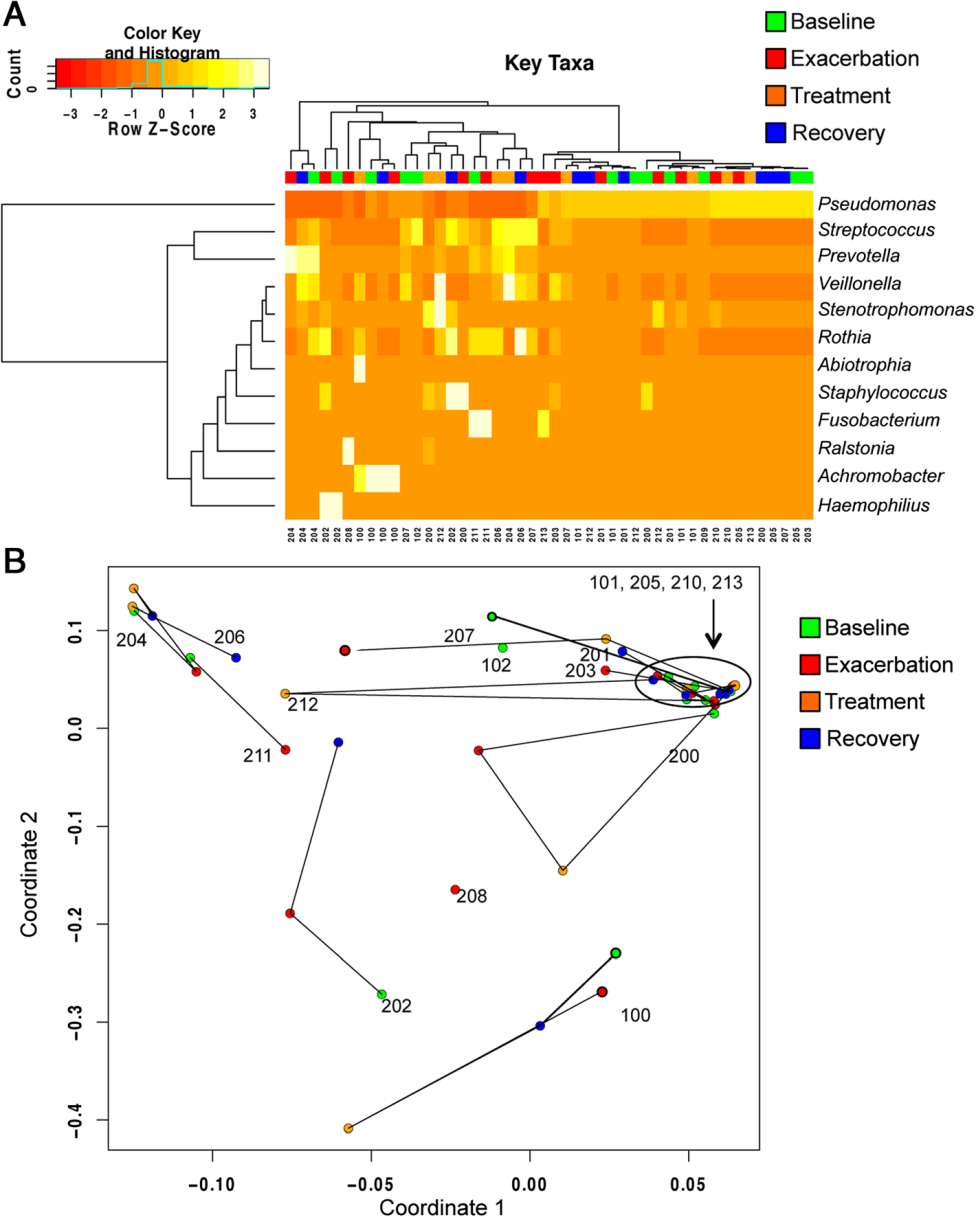
**Microbial communities cluster by patient, not by clinical stage. (A)** Hierarchical clustering of top 12 genera found in patient samples. Each clinical BETR stage is designated by color (baseline, green; exacerbation, red; treatment, orange; recovery, blue) along the top of the diagram; patient number is given across the bottom. The relative abundance for each genus is colored in shades of red (low relative abundance) to yellow or bright white (high relative abundance) as shown in the color key (upper left). The *x*-axis of the color key (row Z-score) indicates the number of standard deviations from the mean relative abundance for each genus. The count histogram indicates the mean counts for all data in sample set. **(B)** Principal coordinate analysis of all patients. Clinical stages are represented by colored dots (baseline, green; exacerbation, red; treatment, orange; recovery, blue) and black lines connect the trajectories of each patient’s microbiome throughout the study. BETR, baseline, exacerbation, treatment and recovery.

Assessing the sputum communities in our cohort, we did not find any evidence of a shift in microbial structure at exacerbation (Figure 2A). Instead, we found that sputum communities in a given patient are more similar to each other than they are to other patients’ communities at the same clinical stage. We confirmed this observation by complete hierarchical clustering and principal coordinate analysis (PCoA; Figure 3).

The relative abundance of *Pseudomonas* drives most of the clustering in our cohort (Figure 3A). Samples dominated by high levels of *Pseudomonas* are shown at the right of the top dendrogram and those by low and medium levels of *Pseudomonas* are at the left and center. Hierarchical clustering analysis revealed no evidence for clustering by clinical stage (Figure 3A). Colored blocks indicate clinical stages across the top of the heat map. When an additional analysis of community similarity (PCoA, Bray-Curtis) was employed to characterize the population of all patients in this study, we again observed no clustering of clinical stages (Figure 3B). Consistent with the hierarchical clustering analysis, a majority of the difference in the population is described by the amount of *Pseudomonas* in the sample. The patient datasets circled on the right are all dominated by *Pseudomonas* (Figure 3B and Additional file 2: Table S2).

In the majority of patients that have a baseline and recovery sample, PCoA revealed that the populations either changed very little (Patients 101, 201 and 205) or that the recovery sample circles back to the baseline sample (Patients 100, 200, 204 and 212). These patterns indicate that while transient changes may occur during exacerbation (most often at the treatment stage), the populations return to their pre-exacerbation composition. Patient 204 and 206’s sputum communities are distinct from the majority of patients as they are dominated by *Prevotella* and *Streptococcus*. For Patient 204, who provided all four BETR samples, the recovery sample is very similar to the baseline sample despite the unusual community of this patient, again emphasizing the theme of stability in sputum bacterial communities.

An exception to the theme outlined above occurred in Patient 202. For Patient 202, the recovery sample did not circle back to its pre-exacerbation state, but interestingly, it moved closer to the majority of samples in the PCoA plot. Patient 202’s dominant genus at baseline and exacerbation stages was *Haemophilus*, a genus that has a lower abundance/prevalence ratio among all other samples analyzed, which was then replaced post-treatment during the recovery phase with *Staphylococcus*, *Streptococcus* and *Pseudomonas*, genera that have higher abundance/prevalence ratios (Figure 2B). Thus, patient 202’s unusual sputum bacterial community was replaced by a community more common to this and other reported cohorts [11,17-19,23,32]. Taken together, hierarchical clustering and principal coordinate analysis show that while there are detectable changes to the sputum microbiome for certain individuals, there is no one genus or pattern of genera correlated with exacerbations across all patients.

### Diversity does not correlate with clinical stage

**Figure 4:**
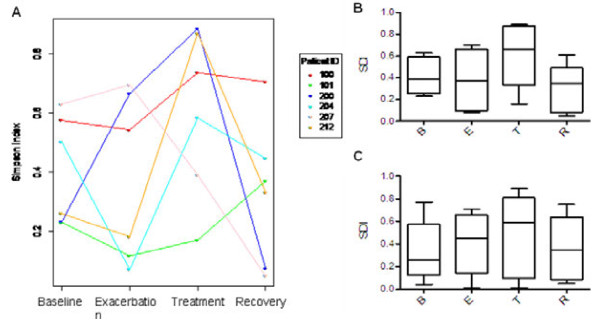
**Diversity does not correlate with clinical stage. (A)** Simpson diversity index for the six patients with samples from all four stages (BETR) are color-coded. A mixed-effect linear model with treatment status as a categorical variable was used to identify significant differences in diversity as a function of clinical status. Analysis of variance showed no significant association between diversity and status. Aggregated Simpson diversity index (SDI) of the six patients with all four BETR samples **(B)** or from all seventeen patients **(C)**. There is no statistically significant difference between stages for patients with all four BETR samples (ANOVA, Tukey post-test, *P* values all >0.05). Paired *t*-test for all patients reveals no statistical difference between any stage (*P* > 0.05). B, baseline; BETR, baseline, exacerbation, treatment and recovery; E, exacerbation; R, recovery; SDI, Simpson diversity index; T, treatment.

Reduced bacterial diversity in sputum has been correlated with decreasing lung function as measured by FEV_1_[11,16-20,22]. Nevertheless, it appears that a decrease in bacterial diversity is a poor predictor of acute exacerbations [11,19]. We calculated the Simpson diversity index (SDI), a measure of the diversity of a sample, for each of the six patients that have a complete BETR dataset (Figure 4A and B) and for all patients (Figure 4C). An SDI of 0 indicates no diversity; an SDI of 1 indicates maximum diversity. Diversity fluctuates dramatically within individual patients (Figure 4A). No obvious pattern emerges that distinguishes one stage from the next when diversity indices are examined on a patient-by-patient basis (Figure 4A). Analysis of variance showed no significant association between diversity and status when data were fitted with a mixed-effect linear model or when aggregated (Figure 4B, six patients with all four BETR samples, ANOVA, Tukey post-test *P* > 0.05; Figure 4C, all patients, paired *t*-test *P* > 0.05). Thus, while reduced diversity is correlated with decreasing lung function over a lifetime, on the short-term timescale of an acute exacerbation, bacterial diversity is not a reliable predictor of an exacerbation.

### No individual genus is predictive of clinical stage transition

**Figure 5:**
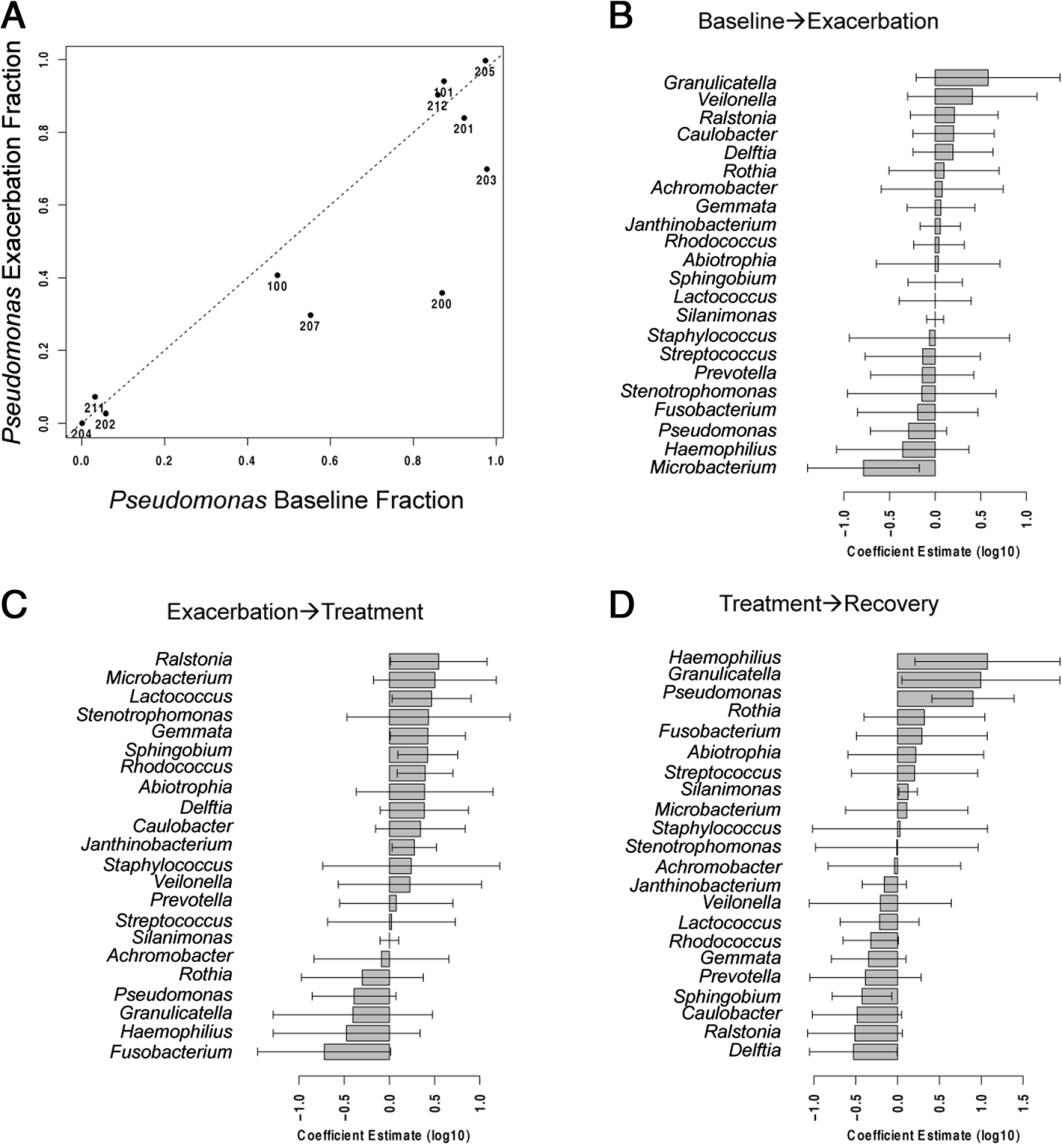
**Changes in individual bacterial abundance at each clinical transition. (A)** Relative abundance of *Pseudomonas* at baseline and exacerbation. Each point represents a different patient labeled with patient ID. The dotted line has a slope of 1. Points to the left of the 1 line indicate an increase in *Pseudomonas* at exacerbation compared to baseline; points to the right of the 1 line indicate a decrease in *Pseudomonas*. Coefficient estimates of each genus for **(B)** baseline to exacerbation, **(C)** exacerbation to treatment and **(D)** treatment to recovery. The top 12 genera from Figure 2 and genera with coefficient estimate significantly different than 1 (*P* < 0.05) are shown. Bars indicate mean coefficient estimate; error bars indicate 95% confidence interval of the mean.

Hierarchical clustering and principal coordinate analysis, as multivariate statistics, do not readily identify changes in individual genera that might be associated with clinical transitions. Thus, we employed a mixed-effects linear model with clinical stage as a categorical fixed effect and patient as a random effect to identify significant differences in the abundance of genera as a function of clinical stage. For example, the relative abundance of *Pseudomonas* at baseline is plotted against its relative abundance at exacerbation (Figure 5A). *Pseudomonas* decreases at the transition from baseline to exacerbation for many patients (points to the right of the dotted line with a slope of 1). To determine if this apparent decrease in *Pseudomonas* at exacerbation (and any other observed change in relative abundance for each genus at each clinical transition) was significant, a mixed-effects linear model, with clinical stage as a categorical main effect, was used to fit these data and calculate the coefficient estimate of the model.

The coefficient estimates of the top 12 genera (including *Pseudomonas*) and any other genus that has a coefficient estimate significantly different from 1 (*P* < 0.05) as they transition between clinical stages are shown in Figure 5B,C,D (bars). However, because we tested many genera simultaneously, we cannot consider these results to be predictive without an additional test of statistical significance, as *P*-value = 0.05, by definition, yields a false positive result 5% of the time under the null hypothesis. For example, with our dataset of roughly 170 genera, we would expect about 9 genera to reach a significance of *P* = 0.05 under the null hypothesis. A Bonferroni correction of *P* values to address the family-wise error rate would require a given genus to achieve a *P* value of less than 0.0003 to reach significance, eliminating all genera from further consideration.

## Discussion

We followed 17 adult CF patients as they transitioned through the BETR stages of a clinical exacerbation. Overall, we found that the sputum microbiome is distinct and resilient within patients throughout time, including over the course of exacerbation and antibiotic treatment. We found no statistically significant difference in absolute bacterial abundance (Table 1, Figure 1A), absolute abundance of *Pseudomonas* (Figure 1B) or composition (Figure 3) between clinical stages within each patient. The majority of patients in our cohort had sputum communities whose dominant members were unperturbed by clinical exacerbation, and these communities appeared to change very little from stage to stage (Figures 2A and 3B). Interestingly, for a patient whose baseline and recovery samples were strikingly different (202), the microbial composition changed to a community more common to this cohort and previously reported cohorts during the course of this study (Figure 3B) [11,17-19,22,23]. Patient 202 received a unique antibiotic cocktail of colistin, meropenem and tobramycin and was the only patient in this cohort to receive colistin (Additional file 1: Table S1). It is possible that colistin contributed to the dissimilarity of Patient 202’s baseline and recovery samples, however, testing this hypothesis would require additional patient samples. Diversity in this cohort fails to be a predictor of clinical exacerbation (Figure 4), which agrees with three recent studies [11,19,20]. We did not observe the previously reported significant [11] or modest [19] decrease in diversity at the treatment stage, possibly due to our small sample size and short timescale. On a patient-by-patient level, we see wide variation in diversity (Figure 4A), which is masked when diversity indices are considered in the aggregate (Figure 4B and C), highlighting the complex, individualized nature of CF sputum communities. We examined how the relative abundance of each detectable organism changes between each clinical stage in each patient and were unable to identify any one genus that signaled a change in clinical status (Figure 5). Importantly, the stability of sputum communities observed throughout exacerbation and antibiotic treatment (Figures 1 and 2A) suggests that a single sputum sample is a consistent and reliable method for sampling the bacteria in CF airways. Sequencing a patient’s sputum microbiome may therefore be a cost effective tool for physicians to create a more complete picture of all bacteria in a patient’s airways, which may potentially inform treatment strategies in the clinic.

We were unable to identify a genus or group of genera that herald a change in clinical stage when the entire community was considered (Figure 3) or when each genus was examined one by one (Figure 5). Assessing diversity on a patient-by-patient basis (Figure 4A) and bacterial abundance on a genus-by-genus basis (Figure 5) as we report here highlights the complexity of identifying a microbial signature of clinical stage in adult CF patients. Identifying the microbial factors that influence clinical transition may require monitoring the long-term dynamics of the airway microbiome of many CF patients over several cycles of exacerbation, such as reported by Zhao *et al*. [11], to tease out the microbial factors that are predictive or indicative of exacerbations. The individualized nature of our cohort’s sputum bacterial communities suggests that a personalized analysis for each patient may be necessary for such predictive power. It is possible that the current resolution of sequencing is not high enough to identify the microbial contribution to exacerbation onset. Higher resolution sequencing to the species or strain level and/or transcriptional profiling of the bacteria in CF sputum may be required to identify how the microbiome contributes to exacerbations. Such analyses may reveal changes in the ratios of virulent and less virulent strains or species, which may drive acute and chronic infections, respectively, without changing the overall load of that genus. For instance, CF patients are known to harbor multiple strains of *P. aeruginosa* in their lungs and over time, virulence factors are selected against, while antibiotic resistance increases [33]. It is possible that exacerbations are caused by a shift of relative abundance from a less virulent strain to a more virulent one during exacerbations. Additionally, Surette and colleagues correlated the presence of *Streptococcus milleri* group (SMG) with poor patient outcomes [34]. Perhaps the ratio of SMG increases at exacerbation and decreases at treatment without changing the overall relative abundance of streptococci in the sputum. Therefore, in addition to higher resolution population level analyses, focusing studies on the interspecies interactions of these relatively few highly abundant and highly prevalent organisms, particularly the response of these organisms to antibiotics, has potential to be informative in the treatment of the polymicrobial infections of the CF airway.

## Conclusions

The results presented here show no microbial signature specific to any clinical stage by any measure as a group or for individual patients. Instead, we found that the sputum microbiome is distinct and resilient within patients throughout time and the stresses of an exacerbation and antibiotic treatment. These data support previous findings that illustrate the complex microbial environment that exists in the sputum obtained from CF airways and support previous work demonstrating that transition to a pulmonary exacerbation is not due to the simple increase in bacterial load or bloom of any one genus or group of genera [11-19,21-23,32]. The individualized nature of our cohort’s sputum bacterial communities make it difficult to identify general trends from a single round of exacerbation. In addition to analyzing the sputum microbiome from sequential rounds of exacerbation, higher resolution sequencing to the species or strain level and/or transcriptional profiling of the bacteria in CF sputum may reveal microbial factors that influence clinical transition. Identifying these clinical transition factors has potential to inform therapeutic strategies to better treat the polymicrobial infections of the CF airway.

## Abbreviations

B: baseline; BAL: bronchial alveolar lavage; BER: baseline, exacerbation and recovery; BETR: baseline, exacerbation, treatment and recovery; CF: cystic fibrosis; CFF: Cystic Fibrosis Foundation; CFPE: cystic fibrosis pulmonary exacerbation; CFTR: cystic fibrosis transmembrane conductance regulator; E: exacerbation; gDNA: genomic DNA; IV: intravenous; NIH: National Institutes of Health; PCoA: principal coordinate analysis; PCR: polymerase chain reaction, qPCR, quantitative PCR; R: recovery; SDI: Simpson diversity index; SMG: *Streptococcus milleri* group; T: treatment; TRC: Translational Research Core.

## Competing interests

The authors declare that they have no competing interests.

## Authors’ contributions

KEP performed the qPCR analysis. THH performed the statistical analyses. ELD prepared all sputum samples for deep-sequencing analysis. HGM and MLS performed the deep-sequencing analysis. AHG, DAH and GAO contributed to study design. KEP, THH and GAO wrote the manuscript. All authors read and gave approval to the final manuscript.

## Supplementary Material

Additional file 1: Table S1 Summarized clinical data of the patients analyzed in this study, including coded the identifier used to label the sample, dates of each clinical event, forced expiratory volume in 1 second (FEV_1_) at each clinical event and the antibiotics used for treatment of the exacerbation event. Click here for file

Additional file 2: Table S2 Raw reads generated from deep sequencing of the V4-V6 region of the 16S rRNA gene. The first column indicates the individual genera identified in at least one sample analyzed in the study, and each subsequent column lists the number of reads for each genus in the sample analyzed. The labeling of each column corresponding to the labeling scheme used in Figure 2A. An entry of 0 indicates no reads for that particular genus in that sample. Click here for file

## References

[B1] Dinwiddie R Pathogenesis of lung disease in cystic fibrosis Respiration 2000 1 3 8 10.1159/000029453 10705255

[B2] Marks MI Clinical significance of *Staphylococcus aureus* in cystic fibrosis Infection 1990 1 53 56 10.1007/BF01644186 2107147

[B3] Stone A Quittell L Zhou J Alba L Bhat M DeCelie-Germana J Rajan S Bonitz L Welter JJ Dozor AJ Gherson I Lowy FD Saiman L *Staphylococcus aureus* nasal colonization among pediatric cystic fibrosis patients and their household contacts Pediatr Infect Dis J 2009 1 895 899 10.1097/INF.0b013e3181a3ad0a 20135845

[B4] Govan JR Nelson JW Microbiology of lung infection in cystic fibrosis Br Med Bull 1992 1 912 930 1281036 10.1093/oxfordjournals.bmb.a072585

[B5] Boucher RC New concepts of the pathogenesis of cystic fibrosis lung disease Eur Respir J 2004 1 146 158 10.1183/09031936.03.00057003 14738247

[B6] Goss CH Burns JL Exacerbations in cystic fibrosis: 1: epidemiology and pathogenesis Thorax 2007 1 360 367 10.1136/thx.2006.060889 17387214 PMC2092469

[B7] Costerton JW Cystic fibrosis pathogenesis and the role of biofilms in persistent infection Trends Microbiol 2001 1 50 52 10.1016/S0966-842X(00)01918-1 11173226

[B8] Heijerman H Infection and inflammation in cystic fibrosis: a short review J Cyst Fibros 2005 1 Suppl 2 3 5 15970469 10.1016/j.jcf.2005.05.005

[B9] Lyczak JB Cannon CL Pier GB Lung infections associated with cystic fibrosis Clin Microbiol Rev 2002 1 194 222 10.1128/CMR.15.2.194-222.2002 11932230 PMC118069

[B10] Harris JK De Groote MA Sagel SD Zemanick ET Kapsner R Penvari C Kaess H Deterding RR Accurso FJ Pace NR Molecular identification of bacteria in bronchoalveolar lavage fluid from children with cystic fibrosis Proc Natl Acad Sci USA 2007 1 20529 20533 10.1073/pnas.0709804104 18077362 PMC2154465

[B11] Zhao J Schloss PD Kalikin LM Carmody LA Foster BK Petrosino JF Cavalcoli JD VanDevanter DR Murray S Li JZ Young VB LiPuma JJ Decade-long bacterial community dynamics in cystic fibrosis airways Proc Natl Acad Sci USA 2012 1 5809 5814 10.1073/pnas.1120577109 22451929 PMC3326496

[B12] Sibley CD Grinwis ME Field TR Parkins MD Norgaard JC Gregson DB Rabin HR Surette MG McKay agar enables routine quantification of the *'Streptococcus milleri’* group in cystic fibrosis patients J Med Microbiol 2010 1 534 540 10.1099/jmm.0.016592-0 20093379

[B13] Field TR Sibley CD Parkins MD Rabin HR Surette MG The genus *Prevotella* in cystic fibrosis airways Anaerobe 2010 1 337 344 10.1016/j.anaerobe.2010.04.002 20412865

[B14] Zemanick ET Wagner BD Sagel SD Stevens MJ Accurso FJ Harris JK Harris JK Reliability of quantitative real-time PCR for bacterial detection in cystic fibrosis airway specimens PLoS One 2010 1 e15101 10.1371/journal.pone.0015101 21152087 PMC2994853

[B15] Huang YJ Lynch SV The emerging relationship between the airway microbiota and chronic respiratory disease: clinical implications Expert Rev Respir Med 2011 1 809 821 10.1586/ers.11.76 22082166 PMC3359942

[B16] Daniels TW Rogers GB Stressmann FA van der Gast CJ Bruce KD Jones GR Connett GJ Legg JP Carroll MP Impact of antibiotic treatment for pulmonary exacerbations on bacterial diversity in cystic fibrosis J Cyst Fibros 2013 1 22 28 10.1016/j.jcf.2012.05.008 22717533

[B17] Stressmann FA Rogers GB van der Gast CJ Marsh P Vermeer LS Long-term cultivation-independent microbial diversity analysis demonstrates that bacterial communities infecting the adult cystic fibrosis lung show stability and resilience Thorax 2012 1 867 873 10.1136/thoraxjnl-2011-200932 22707521

[B18] Filkins LM Hampton TH Gifford AH Gross MJ Hogan DA Sogin ML Morrison HG Paster BJ O'Toole GA Prevalence of streptococci and increased polymicrobial diversity associated with cystic fibrosis patient stability J Bacteriol 2012 1 4709 4717 10.1128/JB.00566-12 22753064 PMC3415522

[B19] Fodor AA Klem ER Gilpin DF Elborn JS Boucher RC Tunney MM Wolfgang MC The adult cystic fibrosis airway microbiota is stable over time and infection type, and highly resilient to antibiotic treatment of exacerbations PLoS One 2012 1 e45001 10.1371/journal.pone.0045001 23049765 PMC3458854

[B20] Zemanick ET Harris JK Wagner BD Robertson CE Sagel SD Stevens MJ Accurso FJ Laguna TA Inflammation and airway microbiota during cystic fibrosis pulmonary exacerbations PLoS One 2013 1 e62917 10.1371/journal.pone.0062917 23646159 PMC3639911

[B21] Stressmann FA Rogers GB Marsh P Lilley AK Daniels TW Carroll MP Hoffman LR Jones G Allen CE Patel N Forbes B Tuck A Bruce KD Does bacterial density in cystic fibrosis sputum increase prior to pulmonary exacerbation? J Cyst Fibros 2011 1 357 365 10.1016/j.jcf.2011.05.002 21664196

[B22] Cox MJ Allgaier M Taylor B Baek MS Huang YJ Daly RA Karaoz U Andersen GL Brown R Fujimura KE Wu B Tran D Koff J Kleinhenz ME Nielson D Brodie EL Lynch SV Airway microbiota and pathogen abundance in age-stratified cystic fibrosis patients PLoS One 2010 1 e11044 10.1371/journal.pone.0011044 20585638 PMC2890402

[B23] Lim YW Schmieder R Haynes M Willner D Furlan M Youle M Abbott K Edwards R Evangelista J Conrad D Rohwer F Metagenomics and metatranscriptomics: windows on CF-associated viral and microbial communities J Cyst Fibros 2013 1 154 164 10.1016/j.jcf.2012.07.009 PMC3534838 22951208

[B24] Gifford AH Moulton LA Dorman DB Olbina G Westerman M Parker HW Stanton BA O'Toole GA Iron homeostasis during cystic fibrosis pulmonary exacerbation Clin Transl Sci 2012 1 368 373 10.1111/j.1752-8062.2012.00417.x 22883617 PMC3419499

[B25] Visualization and Analysis of Microbial Population Structures [http://vamps.mbl.edu]

[B26] NCBI website [http://www.ncbi.nlm.nih.gov/sra]

[B27] Maeda H Fujimoto C Haruki Y Maeda T Kokeguchi S Petelin M Arai H Tanimoto I Nishimura F Takashiba S Quantitative real-time PCR using TaqMan and SYBR green for *Actinobacillus actinomycetemcomitans, Porphyromonas gingivalis, Prevotella intermedia, tetQ* gene and total bacteria FEMS Immunol Med Microbiol 2003 1 81 86 10.1016/S0928-8244(03)00224-4 14557000

[B28] Horz HP Vianna ME Gomes BP Conrads G Evaluation of universal probes and primer sets for assessing total bacterial load in clinical samples: general implications and practical use in endodontic antimicrobial therapy J Clin Microbiol 2005 1 5332 5337 10.1128/JCM.43.10.5332-5337.2005 16208011 PMC1248440

[B29] Warnes GR gplots: Various R programming tools for plotting data. R package version 3.0.1 2013 2013 1 2013 [http://cran.r-project.org/web/packages/gplots/]

[B30] Stacklies W Redestig H Scholz M Walther D Selbig J pcaMethods – a bioconductor package providing PCA methods for incomplete data Bioinformatics 2007 1 1164 1167 10.1093/bioinformatics/btm069 17344241

[B31] Oksanen J Blanchet FG Kindt R Legendre P Minchin PR O’Hara RB Simpson GL Solymos P Stevens MHH Wagner H R package Version 2.0-5 2012 Vegan: Community Ecology Package

[B32] van der Gast CJ Walker AW Stressmann FA Rogers GB Scott P Daniels TW Carroll MP Parkhill J Bruce KD Partitioning core and satellite taxa from within cystic fibrosis lung bacterial communities ISME J 2011 1 780 791 10.1038/ismej.2010.175 21151003 PMC3105771

[B33] Smith EE Buckley DG Wu Z Saenphimmachak C Hoffman LR D'Argenio DA Miller SI Ramsey BW Speert DP Moskowitz SM Burns JL Kaul R Olson MV Genetic adaptation by *Pseudomonas aeruginosa* to the airways of cystic fibrosis patients Proc Natl Acad Sci USA 2006 1 8487 8492 10.1073/pnas.0602138103 16687478 PMC1482519

[B34] Sibley CD Parkins MD Rabin HR Duan K Norgaard JC Surette MG A polymicrobial perspective of pulmonary infections exposes an enigmatic pathogen in cystic fibrosis patients Proc Natl Acad Sci USA 2008 1 15070 15075 10.1073/pnas.0804326105 18812504 PMC2567494

